# Myelodysplastic Syndromes and Modalities of Treatment: An Updated Literature Review

**DOI:** 10.7759/cureus.20116

**Published:** 2021-12-02

**Authors:** Diana I Zamora, Gautami S Patel, Idan Grossmann, Kevin Rodriguez, Mridul Soni, Pranay K Joshi, Saawan C Patel, Devarashetty Shreya, Ibrahim Sange

**Affiliations:** 1 General Medicine, Universidad de Ciencias Médicas, San José, CRI; 2 Internal Medicine, Pramukhswami Medical College, Karamsad, IND; 3 Research, Medical University of Silesia, Faculty of Medical Sciences in Katowice, Katowice, POL; 4 Research, Universidad Americana Facultad de Medicina, Managua, NIC; 5 Research, Shri Lal Bahadur Shastri Government Medical College, Mandi, IND; 6 Department of Medicine, B.J. Medical College, Ahmedabad, IND; 7 Medicine, Pramukhswami Medical College, Karamsad, IND; 8 Internal Medicine, Gandhi Medical College, Secunderabad, Hyderabad, IND; 9 Research, K.J. Somaiya Medical College, Mumbai, IND

**Keywords:** critical anemia, blast cell, acute myeloblastic leukemia, malignant hematology, myelodysplastic syndromes

## Abstract

Myelodysplastic syndromes (MDS) represent a large group of rare and diverse clonal stem cell disorders. These are classified into several different phenotypes and typically arise following a multistep genetic process, whereby genetic mutations alter the DNA damage and cellular stress responses, impacting transcription, RNA splicing, epigenetics, and cytokine signaling. However, despite the advances made regarding molecular pathophysiology and prognostic criteria and the influx of new treatment modalities, management is primarily based on prognostic scores, such as the Revised International Prognostic Scoring System. This poses a significant challenge to current healthcare professionals due to poor comprehension of the underlying pathophysiology. Hence, this review integrates the latest research and treatment modalities for MDS and discusses the different genetic mutations outlined in the revised World Health Organization 2016 MDS classification system and the associated treatment modalities. Additionally, future directions of research and clinical management of MDS are discussed.

## Introduction and background

Myelodysplastic syndromes (MDS) are a group of rare and malignant clonal stem cell disorders. These are classified as hematologic malignancies caused by ineffective hematopoiesis, resulting in morphologic dysplasia, bone marrow failure, and high-risk transformation to acute myeloid leukemia (AML) [1]. This typically arises secondary to a multistep genetic process in which mutations affecting DNA damage and cellular stress responses affect transcription, RNA splicing, epigenetics, and cytokine signaling, which, in turn, leads to the initiation and propagation of malignant clones [2].

In the United States, the annual age-adjusted incidence is estimated at 4 per 100,000 people, with this figure increasing significantly with age [3]. However, in addition to age, several other risk factors have been identified that predispose individuals to MDS, including male gender, obesity, smoking, and prior radiotherapy or chemotherapy. Most cases, nonetheless, are idiopathic [3]. In 2016, the World Health Organization (WHO) created the most current classification system of MDS considering the following five characteristics and new genetic abnormalities: dysplastic lineages, cytopenias, ringed sideroblasts in erythroid elements of bone marrow (BM), blasts, and cytogenetics [4]. The WHO 2016 revision to the MDS classification criteria defined 10 MDS subtypes (Table 1) [4]. In this classification, the presence of *SF3B1* mutation represents MDS-RS even when the ring sideroblast (RS) count is greater than 5-15% [2].

**Table 1:** MDS subtypes defined by the revised WHO 2016 MDS classification criteria [4]. MDS: myelodysplastic syndrome; WHO: World Health Organization

Name	Abbreviation
MDS with single lineage dysplasia	MDS-SLD
MDS with multiple myeloid lineage dysplasia	MDS-MLD
MDS-SLD/MLD with ring sideroblasts	RSS; MDS-MLD-RS
MDS with an excess of blasts	MDS-EB-1
MDS with 10–19% bone marrow and 5–19% blood blasts	MDS-EB-2
MDS with isolated deletion of chromosome 5q (del(5q))	N/A
MDS unclassifiable based on defining cytogenetic abnormality	MDS-U
MDS-U with single lineage dysplasia and pancytopenia	MDS-U-SLD
MDS-U with 1% blood blasts	N/A
MDS with *SF3B1 *with ring sideroblasts count >5–15%	MDS-RS

MDS has long posed a significant therapeutic challenge among healthcare professionals. Despite molecular studies recently redefining the prognostic criteria for MDS, it is largely based on prognostic scores, specifically, the International Prognostic Scoring System Revised (IPSS-R), which divides patients into lower-risk MDS (LR-MDS) and higher-risk MDS (HR-MDS) [5]. This scoring system denotes the following five disease factors: blasts, cytogenetics, hemoglobin, platelet count, and absolute neutrophil count (Table 2). Treatment for LR-MDS focuses on improving cytopenia, particularly anemia, whereas therapy for HR-MDS focuses on delaying disease progression and extending survival [6].

**Table 2:** International IPSS-R for MDS [7]. MDS: myelodysplastic syndrome; IPSS-R: International Prognostic Scoring System Revised

Prognostic factors scored	Risk groups based on total risk score
Percentage of blast cells in bone marrow:	1.5 or less points = very low
Less than or equal to 2 = 0 points	2 to 3 points = low
Greater than 2 to less than 5 = 1 point	3.5 to 4.5 points = intermediate
5 to 10 = 2 points	5 to 6 points = high
Greater than 10 = 3 points	6.5 or more points = very high
Cytogenetics (chromosome changes):	
-Y, del(11q) = 0 points	
Normal, del(5q), del(12p), del(20q), double including del(5q)* = 1 point	
del(7q), +8, +19, i(17q), any other single or double independent clone** = 2 points	
-7, inv(3), +(3q), del(3q), double including -7/del(7q), complex: 3 abnormalities = 3 points	
More than 3 abnormalities = 4 points	
Hemoglobin concentration (g/dL):	
Equal to or greater than 10 = 0 points	
8 to less than 10 = 1 point	
Less than 8 = 1.5 points	
Platelet count ( ×10^9^/L of blood):	
Equal to or greater than 100 = 0 points	
50 to less than 100 = 0.5 points	
Less than 50 = 1 point	
Absolute neutrophil count (×10^9^/L of blood):	
Equal to or greater than 0.8 = 0 points	
Less than 0.8 = 0.5 points	

Advances in massively parallel sequencing technology have enabled the genetic characterization of MDS over the last five years, to the point where the focus has now shifted to translating these findings to improve patient outcomes. Further, in the last 10 years, the Food and Drug Administration has approved the use of three MDS therapeutics, in addition to several groups genetically characterizing MDS to a greater extent than before [8]. The genetic data will assist in diagnosing MDS with equivocal pathologic data and will eventually improve the use of novel and existing drugs, whether they be monotherapies or combination therapies. Moreover, these data will enhance the treatment of MDS patients post-allogeneic bone marrow transplant (BMT) [8].

Due to the influx of new treatment modalities, it is often challenging for hematologists to formulate a management plan for patients based on the aforementioned complex classification. Hence, this review aims to integrate the latest research and treatment modalities for MDS and discuss the different genetic mutations outlined in the revised WHO 2016 MDS classification system and the associated treatment modalities. Additionally, this review will discuss the future directions of research and the clinical management of MDS.

## Review

Genetic mutations and MDS pathophysiology

Molecular abnormalities, such as copy number abnormalities and point mutations, are now identified in most MDS cases due to recent technological and scientific advances [9]. Metaphase cytogenetic analysis detects chromosomal abnormalities in approximately 50% of cases, whereas sensitive techniques, including single-nucleotide polymorphism (SNP) microarrays or array comparative genomic hybridization, detect abnormalities in nearly 80% of patients [10]. More than half of MDS cases are reported to have somatic point mutations, including the vast majority of those with a normal karyotype [11].

These new genetic data have transformed our comprehension of MDS pathophysiology, implicating new biological pathways and providing a new tool for dissecting the complexities of MDS phenotypes. Recurrent mutations have been discovered that affect various critical cellular processes, including RNA splicing, epigenetic regulation of gene expression, DNA damage response, and tyrosine kinase signaling. Specific mutations in these pathways have been linked to distinct morphologic or clinical phenotypes in some cases (Table 3) [10].

**Table 3:** MDS genetic mutations and their impact on cellular processes and pathways. MDS: myelodysplastic syndrome

	Impacted genes/proteins	Processes affected
Spliceosome mutation	*SF3B1*, *SRSF2*, *U2AF1*, and *ZRSR2*	RNA splicing, protein synthesis, mitochondrial dysfunction
Epigenetic regulation of gene expression	*RAS*, *TET2*, and *RNX1*	DNA methylation and histone modifications in gene silencing
DNA damage response	γH2AZ and 53BP1	Nonhomologous end-joining repair mechanisms

RNA splicing mutations predispose many alterations in splicing and are typically early events in MDS pathophysiology, having a significant impact on different genes [12,13]. Collectively, these mutations converge in prevalent dysregulated pathways and cellular processes, including RNA splicing, protein synthesis, and mitochondrial dysfunction (Table 3) [14]. The current literature suggests that splicing factor gene mutations are seen in over 50% of all MDS patients; hence, spliceosome dysfunction is an underpinning driver of MDS pathophysiology [15]. The most frequently mutated splicing factor genes are *SF3B1*, *SRSF2*, *U2AF1*, and *ZRSR2*, which are collectively involved in identifying the 3’ splice sites during the pre-mRNA splicing [16]. However, despite their collective involvement in several cellular processes, these splicing factor genes have different functions. Therefore, following a mutation in any of these genes, a distinct clinical phenotype and prognostic impact in MDS arises [17].

Epigenetic mechanisms and abnormalities, including hypermethylation, underpin the pathophysiology of MDS [18,19]. It is also recognized that epigenetic changes in patients are major drivers of the malignant phenotype of MDS [20]. Hypermethylation of genes that control proliferation and adhesion, such as *RAS*, *TET2*, and *RUNX1*, result in the characteristic features of this condition (Table 3). Few cytogenetic abnormalities have also been identified in high-risk patients, including the deletion of chromosomes 5 and 7, in addition to the isolated deletion of 5q or trisomy 8 [21]. However, despite the enhanced knowledge we now possess regarding MDS and its underlying causes, the molecular mechanisms remain poorly comprehended. It has been suggested that MDS pathogenesis can be attributed to DNA methylation as the disease responds well to drugs that affect this epigenetic change [22].

Alterations to the DNA damage response lead to an increased volume of DNA damage, a critical feature of genetic instability. This factor alone is implicated in the pathogenesis and resulting prognosis of MDS, and it is widely corroborated that the DNA damage response is largely impaired in patients with MDS [23,24]. A study by Popp et al. provided evidence of this using immunofluorescence staining of γH2AZ and 53BP1 to assess DNA damage, specifically double-strand breaks, in MDS cell lines. The findings highlighted that a continuous increase in DNA damage is present across the spectrum of MDS, from low-risk to high-risk patients. The underlying rationale for the colocalization of γH2AZ and 53BP1 can be attributed to the ineffective nonhomologous end-joining repair mechanisms at the site of these double-strand breaks (Table 3) [25].

Genetic mutations and MDS management

The clinical implications of genetic mutations on the management of MDS are widespread, with Kennedy et al. highlighting how DNA sequencing has revolutionized our comprehension of MDS pathogenesis [26]. This has enabled the identification of the genetic mutations previously discussed that are recurrently mutated in myeloid malignancies.

A study by Bejar et al. in 2011 corroborated the involvement of genetic mutations in the management of this disease, concluding that mutations in *TP53*, *EZH2*, *ETV6*, *RUNX1*, and *ASXL1* are predictors of poor prognosis and overall survival in patients. This study adopted several genomic approaches to identify mutations in bone marrow samples obtained from 439 patients with MDS. The results recognized somatic mutations in 18 genes that had previously not been reported as common mutations in these patients. Moreover, it is evident that 51% of patients had a minimum of one point mutation, including 52% of patients who presented with normal cytogenetics [11]. Similar findings were noted by Kim et al. who evaluated similar genetic involvement across a larger cohort of 944 patients with MDS. Here, *TP53*, *EZH2*, *ETV6*, *RUNX1*, and *ASXL1 *were mutated in over 10% of cases; however, this study also identified *SRSF2 *and *DNMT3A *as predictors of poor overall survival [27] (Table 4).

**Table 4:** Associated prognosis with different genetic mutations [27].

Poor prognostic genetic mutations	Good prognostic genetic mutations
SRSF2	SF3B1
DNMT3A	
TP53	
EZH2	
ETV6	
RUNX1	
ASXL1	

Given the variability of survival among patients with MDS, several risk stratification tools, such as the IPSS-R that has been previously discussed, have been developed to influence decision-making (Table 2). These criteria incorporate several aspects of MDS severity, including bone marrow morphology, conventional cytogenetic findings, and the degree of cytopenias [28].

The current literature suggests that somatic mutations are a promising predictor of overall survival in MDS patients, independent of the risk stratification tools and prognostic scoring systems [29]. However, this is refuted because incorporating mutation data alongside clinical and cytogenetic variables provides the most accurate prognostic model, as cytopenias, blast count, and morphology are closely associated with the underlying genetics of the MDS clones [27,30]. Gerstung et al. in 2015 provided evidence for the latter prognostic model, stating that a combination of gene mutation and gene expression data improves the overall outcome prediction in MDS. This study utilized statistical models to interpret the implications of 12 genes that are recurrently mutated in MDS and four cytogenetic alterations on gene expression. The final evaluation also combined diagnostic clinical variables and outcomes in 124 patients with MDS. The results noted that one or more genetic lesions were correlated with the expression levels of an estimated 20% of all genes. This, in turn, accounts for between 20% and 65% of the observed expression variability in these patients [31].

Novel treatment modalities for MDS

Under the latest treatment guidelines, as detailed by the WHO 2016 revised classification criteria, the IPSS-R is a crucial tool in the management of MDS patients [4]. This includes identifying candidates for treatment modalities, specifically as allogeneic hematopoietic stem cell transplantations (ASCT), as this remains the only potentially curative therapeutic approach to MDS. ASCT is primarily reserved for high-risk patients, while treatments such as erythropoiesis-stimulating agents (ESAs) such as epoetin alpha or lenalidomide are predominantly chosen to alleviate symptoms in low-risk patients [32].

ASCT has gained traction in recent years because of its curative potential; however, the findings are still contradictory regarding the target population. Although ASCT is primarily reserved for high-risk patients, a 2017 retrospective analysis suggested that patients with low-risk MDS have a better outcome following ASCT compared to high-risk patients. However, this study did provide a set of criteria that yield the most promising outcome following ASCT, including the selection of the correct source of stem cells, a cytomegalovirus (CMV)-positive donor if the patient is CMV-positive, and utilizing in vivo T-cell depletion results [33].

Despite the previous contradiction, recommendations for ACST from an international expert panel, including representatives from the European Society for Blood and Marrow Transplantation, European LeukemiaNet, Blood and Marrow Transplant Clinical Trial Group, and the International Myelodysplastic Syndromes Foundation, provided clarity on this paradigm. The recommendations support the adoption of ASCT in high-risk IPSS-R patients; however, low-risk IPSS-R patients also meet the eligibility criteria for ASCT if they present with poor-risk genetic features, profound cytopenias, or a high transfusion burden [34]. Additionally, these recommendations emphasize that patients with a significantly high risk score arising from a combination of risk factors have a substantially lower chance of being cured following ASCT. Hence, it is advised that this category of patients be considered for investigational studies [34].

The focus of a large number of investigational studies is the therapeutic agent venetoclax, an oral therapy that inhibits antiapoptotic proteins of the B-cell lymphoma 2 family [35]. A systematic review by Liu et al. evaluated both the efficacy and adverse effects of venetoclax administration in combination with hypomethylating agents in MDS patients. A total of 13 clinical trials were included, involving 1,059 patients. The findings highlight that the incorporation of venetoclax in addition to traditional hypomethylating agents in the treatment of MDS may yield significant clinical benefits. However, the review also noted a possible enhanced risk of developing febrile neutropenia [32,36].

Future research and clinical management

*TP53 *gene mutations are a prevalent finding in patients with MDS, with current studies estimating the presence of *mTP53 *in up to 20% of cases [37]. These patients have a distant molecular phenotype, with a significantly poorer prognosis compared to other genetic mutations; hence, targeting *mTP53 *has been the focus of several clinical trials [38].

APR-246 is a novel therapeutic that has recently completed phase 2 trials. This small molecule selectively induces apoptosis in mTP53 cells through thermodynamic stabilization of the p53 protein [39]. The phase 2 trial recruited 55 patients at a median age of 66 years, with all patients presenting with higher-risk disease, as defined by the IPSS-R. A total of 18 patients discontinued treatment to proceed with ASCT. Moreover, the 30 and 60-day mortality was 2% and 6%, respectively. Collectively, the results of this trial suggest that APR-246 in combination with azacitidine shows promising outcomes; moreover, it is a well-tolerated therapeutic with high response rates in patients with *mTP53* MDS [40].

The most recent clinical trials have focused on risk-adapted and individualized treatment strategies for MDS [41-44]; however, the results of these trials are yet to be published. Future research will evolve from the several novel therapies that are currently being assessed in these clinical trials for the management of MDS. This includes the telomerase inhibitor imetelstat, the oral hypoxia-inducible factor-prolyl hydroxylase inhibitor roxadustat, and the anti-CD-47 antibody magrolimab, to name a few [45-48] (Table 5).

**Table 5:** Population data of recent clinical trials for the management of MDS. MDS: myelodysplastic syndrome; LR: low risk; ESA: erythropoiesis-stimulating agents; AML: acute myeloid leukemia; AZA: azathioprine

Reference	Design	Cases of MDS	Population	Intervention	Conclusion
Platzbecker et al. (2020) [45]	Phase 2/3 trial	38	Transfusion-dependent patients with non-del(5) LR MDS post-ESA therapy	Imetelstat	Imetelstat achieved durable red blood cell transfusion independence. A reduction in variant allele frequency of mutations was also observed in several patients
Chen et al. (2019) [46]	Randomized controlled trial	305	MDS patients who had received dialysis and ESA therapy with epoetin alfa for a minimum of six weeks	Roxadustat	Oral roxadustat did not yield superior results than epoetin alfa therapy for anemia in MDS patients
Sallman et al. (2020) [48]	Phase 1b trial	52	AML patients unfit for intensive chemotherapy	Magrolimab	Magrolimab was both well-tolerated and safe, inducing no immune-related AEs. On-target anemia was mitigated in patients
Swaminathan et al. (2021) [49]	Phase I/II trial	73	Patients of any age who were receiving first-salvage treatment for FLT3-ITD AML or those over the age of 60 years with untreated MDS	Quizartinib with azacitidine	Quizartinib-based combinations proved effective in phase I clinical trials, particularly with AZA for patients with FLT3-ITD-mutated AML

Platzbecker et al. in 2020 reported on the findings of a phase 2 clinical trial that investigated imetelstat in transfusion-dependent subjects with MDS deemed low or intermediate risk by the IPSS-R refractory to ESA treatment. The results determined that imetelstat achieved durable red blood cell transfusion independence. Moreover, a reduction in variant allele frequency of mutations was also observed in several patients. The adverse events reported were both manageable and reversible grade >3 cytopenias, highlighting the safety of this therapeutic. Because of the positive and promising findings of the phase 2 trial, the phase 3 part of this study is currently open for enrollment [45].

Chen et al. in 2019 conducted a randomized controlled trial that investigated roxadustat treatment for MDS patients with anemia who had received dialysis and ESA therapy, with epoetin alfa, for a minimum of six weeks. Patients were randomized to receive either epoetin alfa or roxadustat following six weeks of ESA therapy. The primary endpoint measured was the mean change in hemoglobin level from baseline to the end of the study period at 27 weeks. The findings suggested that roxadustat is equally effective as epoetin alfa therapy for anemia in MDS patients [46].

Sallman et al. in 2020 reported on the results of a phase 1b clinical trial that investigated the combination of magrolimab with azacitidine in AML patients, including *TP53*-mutant AML. A total of 52 patients were enrolled in the trial, with 65% having a *TP53 *mutation. The safety profile of this combination therapy did not reveal any cause for concern, with it being well-tolerated and presenting a similar profile to that of azacitidine monotherapy. The findings of this trial demonstrated that 71% of patients yielded an objective response, 48% achieved complete remission, and 24% presented with stable disease. Collectively, the evidence demonstrates the promising impact of magrolimab as a novel immunotherapy that blocks a key macrophage checkpoint in AML patients. Efficacy is seen across both *TP53*-mutant patients and wild-type AML patients, warranting a phase 3 trial to be conducted. This study is currently ongoing in expansion cohorts (NCT03248479) [48].

Swaminathan et al. in 2021 hypothesized that the combination therapy of quizartinib plus either azacitidine or low-dose cytarabine may improve the outcomes in patients with FMS-like tyrosine kinase 3-internal tandem duplication (FLT3-ITD)-mutated AML. The findings of this phase I/II trial of 73 patients noted that a composite response was achieved in 87%. Moreover, the median overall survival was enhanced in the combination therapy cohort. These results give evidence for the effectiveness of quizartinib as a combination therapy, specifically with azacytidine, in patients with FLT3-ITD-mutated AML [49].

Limitations

The primary limitation of this review is that we could not evaluate all the relevant data regarding future research and novel approaches to the management of MDS as the findings of several clinical trials are yet to be published. Moreover, this review focuses on genetic mutations that underpin the pathogenesis of this condition and the corresponding prognosis and management; however, it does not discuss the management of idiopathic MDS.

## Conclusions

The evidence provided in the studies discussed in this review article highlight that significant variability underpins the prognosis and survival among patients with MDS. Although several risk stratification tools and prognostic measures have been developed, they do not successfully predict the disease progression in the vast majority of cases. The clinical significance of this review article is to establish the association between genetic mutations, pathophysiology, and subsequent management in patients with MDS. These variables are closely associated and should be evaluated in combination during clinical decision-making. We believe that this review article and the evidence provided can serve as a reference when healthcare professionals are faced with challenges regarding MDS management. The differing genetic mutations that result in the various MDS clinical phenotypes were discussed, in addition to identifying the mutations that are predictors of poor prognosis and overall survival in these patients. Lastly, we highlighted the areas of future research that will provide further comprehension of the pathogenesis of MDS and enable risk-adapted and individualized treatment strategies for this condition.
